# Reconstructing the transmission dynamics of rubella in Japan, 2012-2013

**DOI:** 10.1371/journal.pone.0205889

**Published:** 2018-10-17

**Authors:** Masaya M. Saito, Hiroshi Nishiura, Tomoyuki Higuchi

**Affiliations:** 1 The Institute of Statistical Mathematics, Tachikawa, Tokyo, Japan; 2 CREST, Japan Science and Technology Agency, Kawaguchi-shi, Saitama, Japan; 3 Graduate School of Medicine, Hokkaido University, Kita-ku, Sapporo, Japan; University of Florida, UNITED STATES

## Abstract

**Background:**

Japan experienced a nationwide rubella epidemic from 2012 to 2013, mostly in urban prefectures with large population sizes. The present study aimed to capture the spatiotemporal patterns of rubella using a parsimonious metapopulation epidemic model and examine the potential usefulness of spatial vaccination.

**Methodology/Principal findings:**

A metapopulation epidemic model in discrete time and space was devised and applied to rubella notification data from 2012 to 2013. Employing a piecewise constant model for the linear growth rate in six different time periods, and using the particle Markov chain Monte Carlo method, the effective reproduction numbers were estimated at 1.37 (95% CrI: 1.12, 1.77) and 1.37 (95% CrI: 1.24, 1.48) in Tokyo and Osaka groups, respectively, during the growing phase of the epidemic in 2013. The rubella epidemic in 2012 involved substantial uncertainties in its parameter estimates and forecasts. We examined multiple scenarios of spatial vaccination with coverages of 1%, 3% and 5% for all of Japan to be distributed in different combinations of prefectures. Scenarios indicated that vaccinating the top six populous urban prefectures (i.e., Tokyo, Kanagawa, Osaka, Aichi, Saitama and Chiba) could potentially be more effective than random allocation. However, greater uncertainty was introduced by stochasticity and initial conditions such as the number of infectious individuals and the fraction of susceptibles.

**Conclusions:**

While the forecast in 2012 was accompanied by broad uncertainties, a narrower uncertainty bound of parameters and reliable forecast were achieved during the greater rubella epidemic in 2013. By better capturing the underlying epidemic dynamics, spatial vaccination could substantially outperform the random vaccination.

## Introduction

Rubella is a vaccine-preventable viral infectious disease caused by rubella virus. While the infection itself is mostly self-limiting, infection among pregnant women during the early stage of pregnancy (e.g., first trimester) can involve congenital rubella syndrome (CRS), resulting in fetal death, miscarriage or congenital malformations including deafness, cataracts, heart defects and microcephaly. Appropriate prevention by vaccination is therefore crucial; however, limited vaccination coverage is known to induce elevated age at infection, and thus, may contribute to an increased incidence of CRS [1–8]. Japan experienced a nationwide rubella epidemic from 2012 to 2013, which involved many infections among adults with a total of 45 reported CRS cases. While the country has clearly been on its way to achieve sufficient herd immunity to prevent any additional major epidemics, a seroepidemiological analysis of rubella revealed a “pocket” of susceptible individuals among males aged 30–49 years [2].

**Fig 1:**
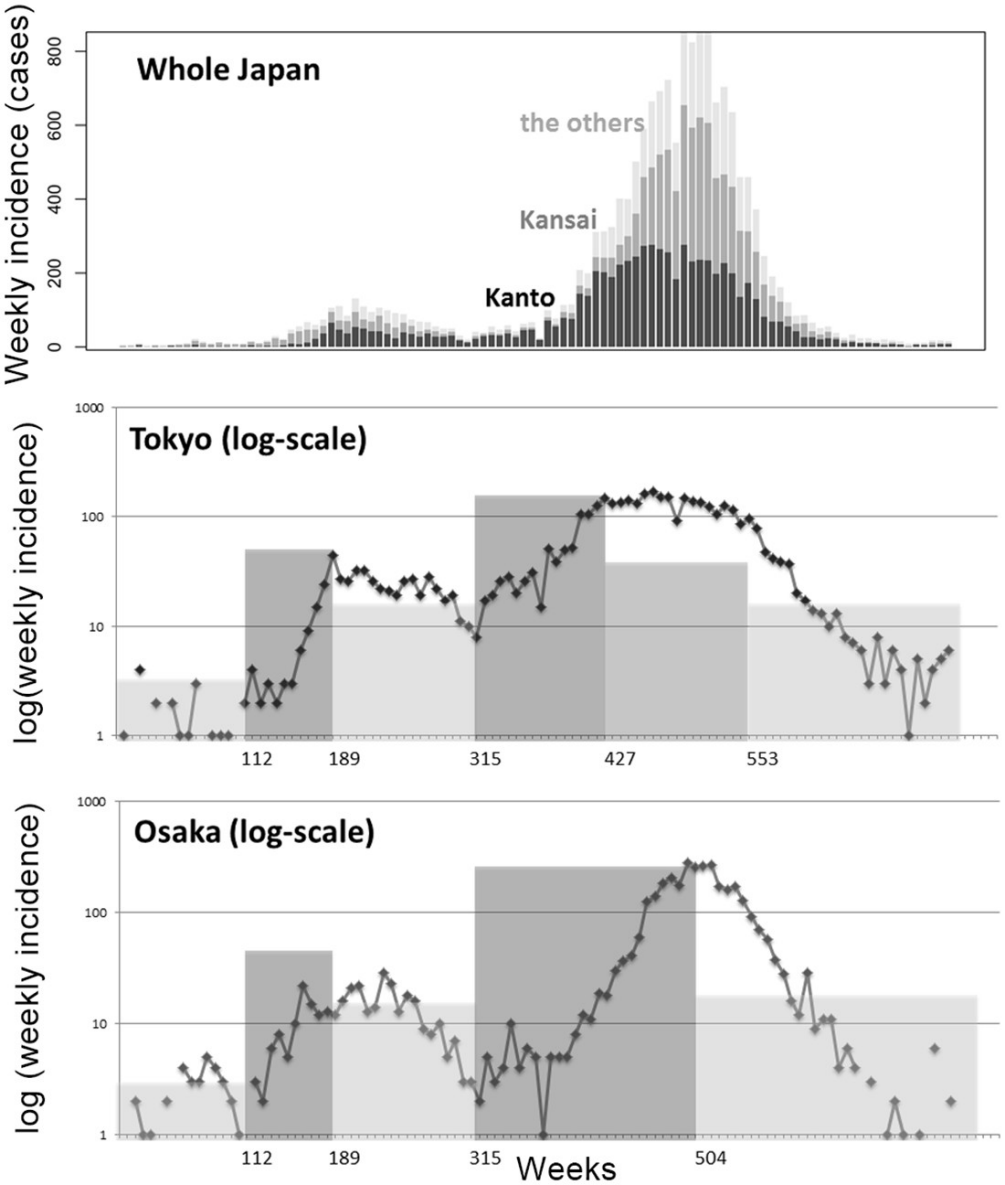
Epidemic curve of the rubella epidemic from 2012 to 2013, Japan. Top: Weekly number of notified cases in Kanto (Tokyo and adjacent prefectures), Kansai (Osaka and adjacent prefectures) and the rest of Japan. Middle and bottom: The number of notified cases in Tokyo (middle) and Osaka (bottom) in log-scale. Epidemic periods are represented by gray-shaded areas, which were visually determined by linear/eyeball extrapolation of the trend, i.e., the epidemic period is switched when the increasing or decreasing trend is swapped.

In addition to age and gender specificities, it must be noted that rubella cases during the 2012–2013 rubella epidemic in Japan have been mostly seen in urban areas. Among the total of 16,730 notified cases, 4,116 (24.6%) and 3,600 (21.5%) were reported from Tokyo and Osaka, the two largest prefectures in Japan. Conversely, the cumulative number of cases during the same period remained below 10 cases in 30 prefectures. Fig 1 shows the epidemic curve from 2012 to 2013. Cases were mostly seen in urban prefectures with large population sizes, and two different patterns of epidemic curves, i.e., the one in (i) Tokyo and adjacent areas and the other in (ii) Osaka and areas connected to Osaka, were identified. Namely, the transmission dynamics was geographically heterogeneous and it is likely that the majority of transmissions have occurred in urban cities associated with either Tokyo or Osaka.

To quantify the spatial heterogeneity of rubella in Japan and incorporating that heterogeneity into future intervention planning, it is beneficial to capture the spatiotemporal dynamics accounting for human mobility. The metapopulation epidemic model is a suitable tool to capture spatiotemporal dynamics over discrete patches, which are in line with the actual spatial distribution of a total of 47 prefectures in Japan [9]. Given that a substantial fraction of adults remains susceptible in the present day population in Japan [2], it is essential to explore possible vaccination strategies. The present study aimed to capture the spatiotemporal patterns of rubella using a parsimonious metapopulation epidemic model and examine the potential usefulness of a vaccination program that focuses on spatial heterogeneity.

## Materials and methods

### Data source

As a notifiable disease in Japan, it is obligatory for rubella to be reported to public health centers and then to the National Institute of Infectious Diseases in Tokyo. Clinical diagnosis rests on the triad of rash, swollen lymph node and fever. We examined the weekly rubella notification data from 2012 to 2013 across all age groups by 47 prefectures.

### Epidemiological model

We employed the so-called susceptible-infectious-recovered (SIR) model in discrete time and space, as originally proposed elsewhere [10]. In this model, the numbers of susceptible, infectious and recovered individuals in week *t* and prefecture *i* are defined as *S*_*i*,*t*_, *I*_*i*,*t*_ and *R*_*i*,*t*_, respectively. Transitions between compartments as a function of time and space are described by

$$\begin{array}{l} S_{i,t+1}=S_{i,t}-\Delta (S_{i,t}\to I_{i,t+1})-\sum_{j\neq i}\Delta (S_{i,t}\to S_{j,t+1})+\sum_{j\neq i}\Delta (S_{j,t}\to S_{i,t+1}),\\ I_{i,t+1}=I_{i,t}+\Delta (S_{i,t}\to I_{i,t+1})-\Delta (I_{i,t}\to R_{i,t+1})-\sum_{j\neq i}\Delta (I_{i,t}\to I_{j,t+1})+\sum_{j\neq i}\Delta (I_{j,t}\to I_{i,t+1}),\\ R_{i,t+1}=R_{i,t}+\Delta (I_{i,t}\to R_{i,t+1})-\sum_{j\neq i}\Delta (R_{i,t}\to R_{j,t+1})+\sum_{j\neq i}\Delta (R_{j,t}\to R_{i,t+1}), \end{array}$$ (1)

where Δ(• → •) are random variables that are determined by the following distribution functions:

$$\begin{array}{l} \Delta (S_{i,t}\to I_{i,t+1})\sim \text{Binom}\left(S_{i,t},1-\text{exp}\left(-\frac{\beta I_{i,t}\Delta t}{N_{i,t}}\right)\right),\\ \Delta (I_{i,t}\to R_{i,t+1})\sim \text{Binom}\left(I_{i,t},1-\text{exp}\left(-\gamma \Delta t\right)\right),\\ \Delta (X_{i,t}\to X_{j,t+1})\sim \text{Binom}\left(X_{i,t},{\tilde{p}}_{i,j}\right)\;\text{for}\;X\in \left\{S,I,R\right\}, \end{array}$$ (2)

where

$$N_{i,t}=S_{i,t}+I_{i,t}+R_{i,t}$$

and

$${\tilde{p}}_{i,j}=1-\text{exp}(-\Delta t\sum_{j}\rho_{ij})\frac{1-\text{exp}(-\Delta t\rho_{ij})}{\sum_{k}1-\text{exp}(-\Delta t\rho_{ik})},$$ (3)

where *ρ*_*ij*_ is the per capita rate of mobility from prefecture *i* to *j* as derived from the statistical survey of regional migration by the Ministry of Land, Infrastructure, Transport and Tourism [11] and Δ*t* is the time unit of our simulation (7 days) which is assumed as equivalent to the mean time to recovery, 1/*γ*. The derivation of Eq (3) can be understood by the discretizing a survival probability of a competing risk model [12]. Table 1 lists variables and parameters used in our model.

**Table 1:** Symbols used in our model.

Symbol	Description
Latent (simulation variables)	
*S*_i,t_	Number of susceptible individuals in prefecture *i* and week *t*
*I*_i,t_	Number of infectious individuals in prefecture *i* and week *t*
*R*_i,t_	Number of recovered individuals in prefecture *i* and week *t*
Observed data	
*J*_i,t_	Observed number of cases in prefecture *i* and week *t*
Fixed parameters	
*ρ*_ij_	Per capita rate of movement from prefecture *i* to *j*
1/*γ*	Mean infectious period (weeks)
*s*_0_	Initial fraction susceptible
Estimated parameters	
*β*_gs_	Transmission rate in geographic group *g* and time period *s*
***R***_g,s_	Effective reproduction number in geographic group *g* and time period *s*

The transmission rate *β* for rubella is assumed to vary with time, perhaps due to seasonality and human contact behaviors. To permit time-dependent *β* to be interpretable, we employed a piecewise constant model for *β*. From Fig 1, we divide the epidemic into five different phases [Days 0–111, 112–188, 189–314, 315–426 (or 315–503) and 427– (or 504–), where Day 0 is 2 January 2012]. The phases were originally determined by eyeball; in each phase, referring to the rubella case counts, we determined that the qualitative pattern is approximated by the piecewise-liner model on the exponential scale with five knots. Seasonality of rubella during pre-vaccination era has not been objectively identified in Japan, and we did not employ any unsupported mathematical functional form for the transmissibility. In the first phase, only a small number of cases, as seen in other years, were observed. Phases (ii) and (iii) were increasing and decreasing phases in 2012, and phases (iv) and (v) were increasing and decreasing phases in 2013, respectively. In Tokyo, an additional time phase of a “sustained” period was considered between (iv) and (v). That is, in Tokyo, we employed phases (iv), (sustained) and (v) from Days 315–426, 427–552 and 553–, respectively. In Osaka, phases (iv) and (v) were considered from Days 315–503 and 504–, respectively. According to that pattern, we divided prefectures into two groups, i.e., those associated with Tokyo and Osaka, and Osaka group included Fukui, Gifu, Mie, Shiga, Kyoto, Osaka, Hyogo, Nara, Wakayama, Yamaguchi, Fukuoka, Saga and Okinawa, and remainders belonged to Tokyo group. The initial count of cases in Tokyo and Osaka at Day 0 was 5, and *I*_*i*,0_ = 0 for the rest. Therefore, *S*_*i*,0_ was assumed as *s*_0_*N*_*i*_, where *s*_0_ is the fraction susceptible in the beginning of the epidemic, assumed as 1/6 [13] (close to an inverse of *R*_0_, the basic reproduction number, that represents the average number of secondary cases generated by a single primary case in a fully susceptible population, which yields the herd immunity threshold, and allow *β* to scale the reproduction number), and *N*_*i*_ is the population size of prefecture *i* (*R*_*i*,0_ = *N*_*i*,0_ − *S*_*i*,0_ − *I*_*i*,0_). Unknown parameters would then be *β* for each phase and space (i.e., six parameters for Tokyo and five parameters for Osaka). Because 1/*γ* = 1 (i.e. the normalized system), the estimates are also interpreted as the effective reproduction number, *R*_*t*_, which indicates the average number of secondary cases generated by a single primary case.

### Statistical estimation

In the abovementioned model, let *J*_i,t_ be the transient counts of cases in week *t* and prefecture *i*, which correspond to Δ(*S*_*i*,*t*_ → *I*_*i*,*t*_) per week (1). Assuming that the cases were independently diagnosed and notified, we assume

$$J_{i,t}\sim \text{Pois}(•;1+\Delta (S_{i,t}\to I_{i,t+1})),$$ (4)

where Pois(•;*λ*) represents Poisson distribution with expected value *λ*. *J*_i,t_ represents the observed data. The likelihood to estimate model parameters using all observed data is

$$\text{lnL}(\text{β};{\text{J}}_{i,t})=\sum_{\text{t}=1}^{\text{104}}\sum_{i=1}^{\text{47}}\text{ln}\int_{\Lambda_{i,t+1}}\text{Pois}(J_{i,t};1+\Lambda_{i,t+1})\text{Binom(}\Lambda_{i,t+1})d\Lambda .$$ (5)

where

$$\Lambda_{i,t+1}=\Delta (S_{i,t}\to I_{i,t+1}).$$ (6)

It should be noted that the Poisson distributed likelihood function was employed in an ad-hoc manner due to a practical difficulty in implementing estimation using an alternative likelihood and filtering method. To be technically precise, a binomially distributed likelihood function might more appropriately capture the observed phenomena, and technical details on this point are described in S1 Text.

A total of 11 parameters for *β* were estimated by using the Markov chain Monte Carlo (MCMC) method, by employing a flat prior distribution and a particle filtering method as well as the Metropolis-Hastings algorithm. In this instance, the posterior distribution is informed primarily for each chain by the likelihood based on particle filtering (or equivalently sequential Monte Carlo approximation) [14]. For each MCMC step, the updated unobserved Δ(*S*_*i*,*t*_ → *S*_*i*,*t*+1_) is used as priors in the Metropolis-Hastings algorithm. The Monte Carlo estimation algorithm is employed for estimating the posterior distribution. This “nested” Monte Carlo approach is referred to as the particle MCMC method, proposed by Andrieu et al. [15]; the method was recently applied to estimate the age-depth relationship of the Dome Fuji ice core in Antarctica [16]. To avoid massive rejection of nearly all proposals due to high values of likelihood by the Metropolis-Hastings algorithm, we employ parallel tempering (see S1 Text for details of parallel tempering). Parallel tempering supplements local configurational Metropolis moves with global “swap” moves that update an entire set of configurations, thereby controlling the tails of posterior distribution. We set the annealing temperature, *T*_pt_, the extent by which likelihood exploration is determined, i.e., to be 16, 32 and 64, among which the value 32 was taken as default. We performed 4,653 + 40,000 iterations of the run of MCMC algorithm. The first 4,653 iterations were discarded as the burn-in period. The convergence of the MCMC is judged by Gelman-Rubin criterion (GRC) [17,18]: each track of the MCMC chain was split into five sub-chains and the GRC was estimated for each parameter. A GRC value close to 1 (conventionally less than 1.1) was regarded as the signature of convergence, and the optimal annealing temperature was selected by GRC.

### Spatial vaccination

Hereafter, the “spatial vaccination” represents a theoretical vaccination scheme that geographically prioritizes urban prefectures with a large population size. Using the parameterized metapopulation model, the effectiveness of spatial vaccination was explored. Specifically, we examined scenarios in which a susceptible fraction among the total population can be vaccinated and reduced by 1%, 3% and 5%, the values of which are in line with the actual amount of vaccines to be potentially secured. These values were examined as the population with these coverages would still remain to be below the epidemic threshold. The vaccine was assumed to elicit all-or-nothing effect and the vaccine efficacy was assumed to be 100% [2]. In our scenario analysis, the total amount of vaccines was pre-fixed in each scenario and assumed to be distributed to different prefectures in advance of the 2012 epidemic. In one scenario, the total volume of vaccines was distributed to Tokyo only. In another scenario, vaccines were distributed to Tokyo and Osaka only. According to the rank of prefectural population size, we varied the number of prefectures to which vaccines are allocated. To do so, we assumed that vaccinated prefectures increase in a descending order of the rank of population size (i.e. prefectures with bigger population sizes were prioritized for vaccination). The most evenly distributed scenario was to equally cover all 47 prefectures.

### Ethical considerations

The present study reanalyzed data that is publicly available in Japan. As such, the datasets used in our study were de-identified and fully anonymized in advance, and the analysis of publicly available data without identity information does not require ethical approval.

### Availability of supporting data

The present study used publicly available data, and essential components of the epidemiological data are available as S1 Table.

## Results

### Parameters governing infectiousness

**Fig 2:**
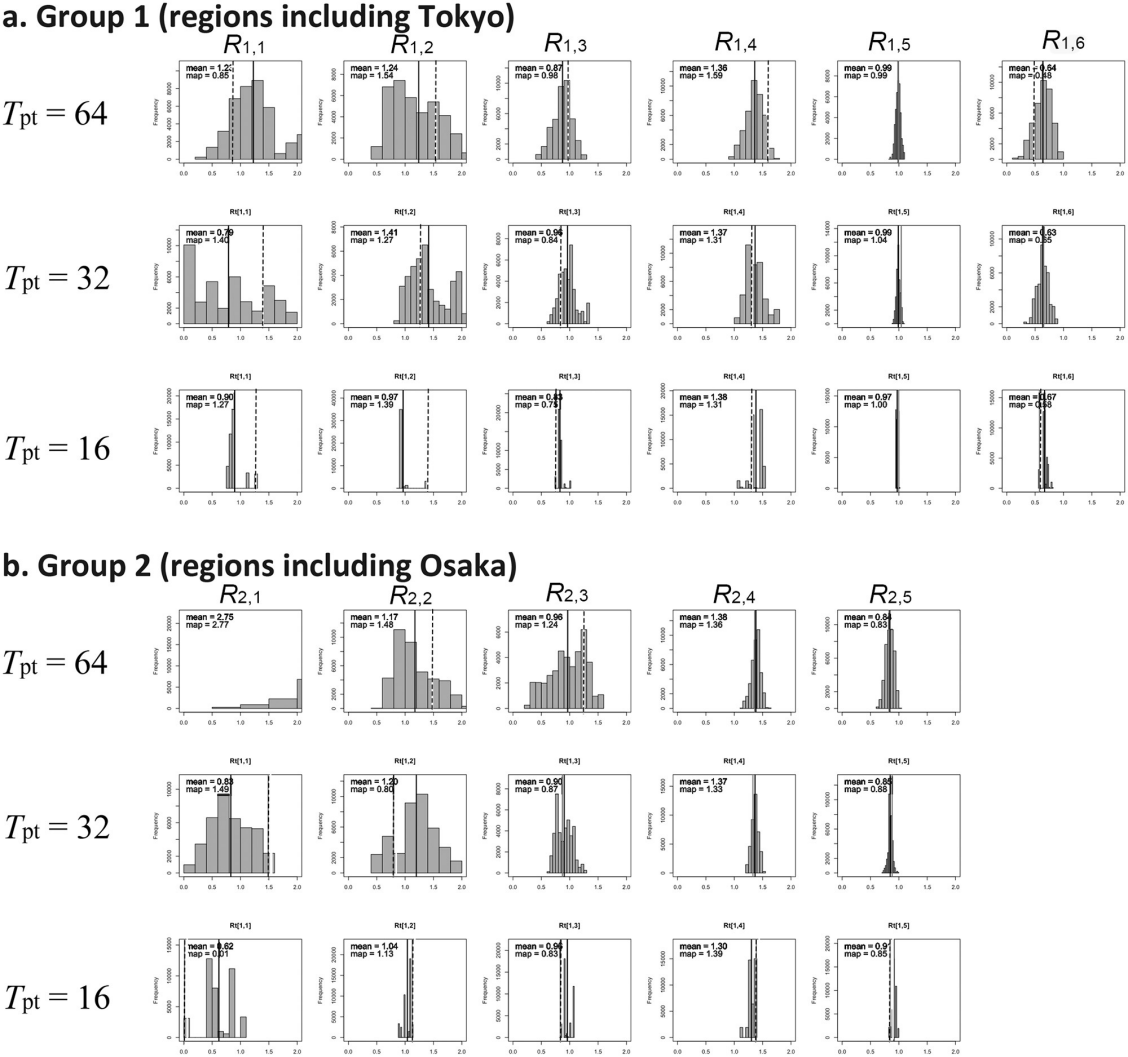
Posterior distributions of the effective reproduction number. Marginal posterior distributions of the effective reproduction numbers are shown for different annealing temperatures (i.e., 16, 32 and 64), as obtained from 40,000 chains. Group 1 represents Tokyo and adjacent prefectures, while group 2 represents Osaka and adjacent prefectures. Tokyo has six panels while Osaka has only five because the epidemic period in Osaka was less complex than Tokyo (see Fig 1). The vertical black line shows the posterior mean value, while the vertical dashed line shows the posterior map (i.e., maximum a posteriori) value, which yields the greatest likelihood value.

Fig 2 shows the sensitivity of our parameter estimates to different annealing temperatures. When the temperature is varied, the expected value of *R*_g,s_ in 2013 did not vary considerably, whereas the variance became smaller with lower temperature, indicating that the estimation was reliable (i.e., reproducible). Conversely, the shape of the posterior distribution for *R*_g,s_ in 2012 was varied by taking lower temperature, reflecting a small number of infected individuals and limited identifiability to estimate *β*_*g*,*s*_ during the corresponding period. S1 Fig shows the limited convergence with annealing temperature 32 given 2012 data only (e.g. R[2.1] and R[2.2]).

**Fig 3:**
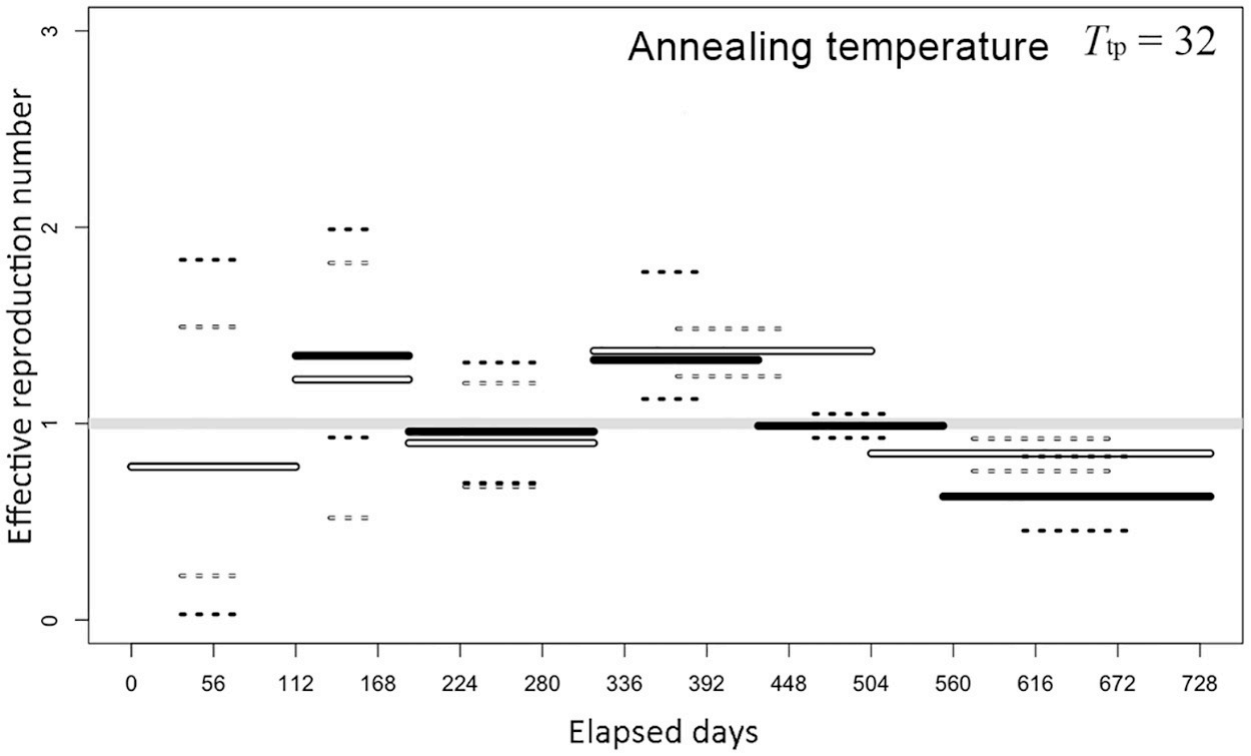
Effective reproduction number of the rubella epidemic from 2012 to 2013, Japan. The posterior mean (continuous line) and 95% credible intervals (CrI; dashed lines) are shown. Black bars represent group 1, i.e., Tokyo and adjacent prefectures, while white bars represent group 2, i.e., Osaka and adjacent prefectures. Monte Carlo particles yielded by the chain with annealing temperature 32 are used for obtaining these estimates. Day 0 is 2 January 2012.

Fig 3 shows time-dependent estimates of transmissibility by group of regions *g* = 1 and 2 (i.e., Tokyo and Osaka groups with the number of prefectures at 34 and 13, respectively) with different time periods (s = 1, 2,…, 6). Transmissibility, as measured by the effective reproduction number, *R*_g,s_, which indicates the average number of secondary cases generated by a single primary case during the specified period, was informed by the estimate of the transmission rate *β*_*g*,*s*_, i.e., *R*_g,s_ = *s*_0_*β*_*g*,*s*_/*γ*, where *s*_0_ represents the initial fraction susceptible. *R*_g,s_ during the growing phase of the 2012 epidemic was estimated at 1.41 (95% CrI: 0.93, 1.99) for the Tokyo group and 1.20 (95% CrI: 0.52, 1.82) for the Osaka group, respectively. Similarly, *R*_g,s_ during the growing phase of the 2013 epidemic was 1.37 (95% CrI: 1.12, 1.77) and 1.37 (95% CrI: 1.24, 1.48), respectively.

### Comparison between observed and predicted epidemics

**Fig 4:**
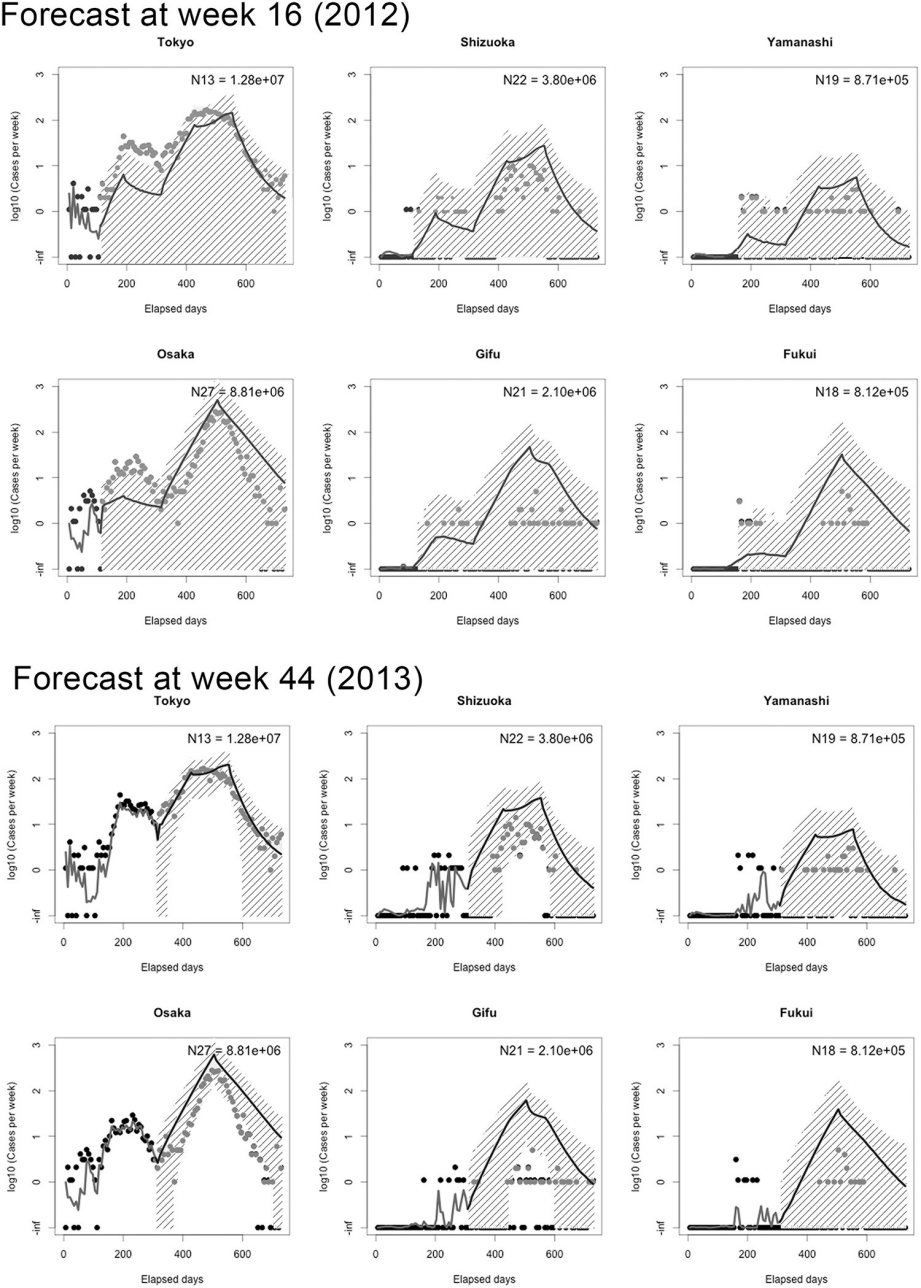
Forecasting rubella epidemics in 2012 and 2013, Japan. Forecasts with 95% prediction intervals for six prefectures are shown. Six prefectures with different population size (each estimate on the upper right corner) are chosen and displayed. For the 2012 forecasting, we repeated the filtering process up to 112th day (when an exponential growth in the first year begins) and repeated the prediction process up to the end of the epidemic. Forecasted weekly notifications are shown in log-scale. Mean and 95%CrIs in filtering process are displayed by solid curves and gray-shaded areas, respectively. For the 2013 forecasting, the filtering process was continued up to 308th day on which an exponential growth in the second year began.

Taking parameter estimates with an annealing temperature of 32, Fig 4 compares the observed and predicted data, the latter of which were generated by filtering the empirical data up to the beginning of *s* = 2 (i.e., week 16) to avoid any uncertainty associated with initial conditions. That is, we sampled parameters from posterior distributions that were obtained up to day 112 and then simulated the epidemics and case reporting using those posterior distributions (see S2 Fig. for the vertical axis taking the absolute number of cases). Due to broad uncertainty of parameters in 2012, the size of the epidemic was highly variable. When we filtered the data by week 44 (i.e., s = 4), then uncertainty was greatly reduced. See S1 Text for technical details of parallel tempering including the detailed procedures in S3 Fig.

To quantitatively compare the performance of prediction by the date at which forecasting is performed, we computed the root mean square error (RMSE) as informed by

$${\text{RMSE}}_{i,t|\tau }:=\int \text{[}{\hat{J}}_{i,t}-J_{i,t}{\text{]}}^{2}p(x_{t};{\left(J_{i,s}\right)}_{1\leq i\leq 47,1\leq s\leq \tau })dx_{t},$$ (7)

where *p*(*a*;*b*) represents the posterior density of *a* given observed data *b*, *x*_*t*_ encompasses all latent states (i.e. SIR model) at time *t*., ${\hat{J}}_{i,t}$ represents the predicted incidence in prefecture *i* at time *t*. Accordingly, this RMSE may also be referred to as the posterior mean squared error. To ease computation of RMSE while having many sample paths (i.e., epidemic trajectories), we computed the following

$${\text{RMSE}}_{i,*|\tau }:={\left(\frac{1}{T-\tau }\sum_{t>\tau }{\text{RMSE}}_{i,t|\tau }^{}\right)}^{1/2}.$$ (8)

**Fig 5:**
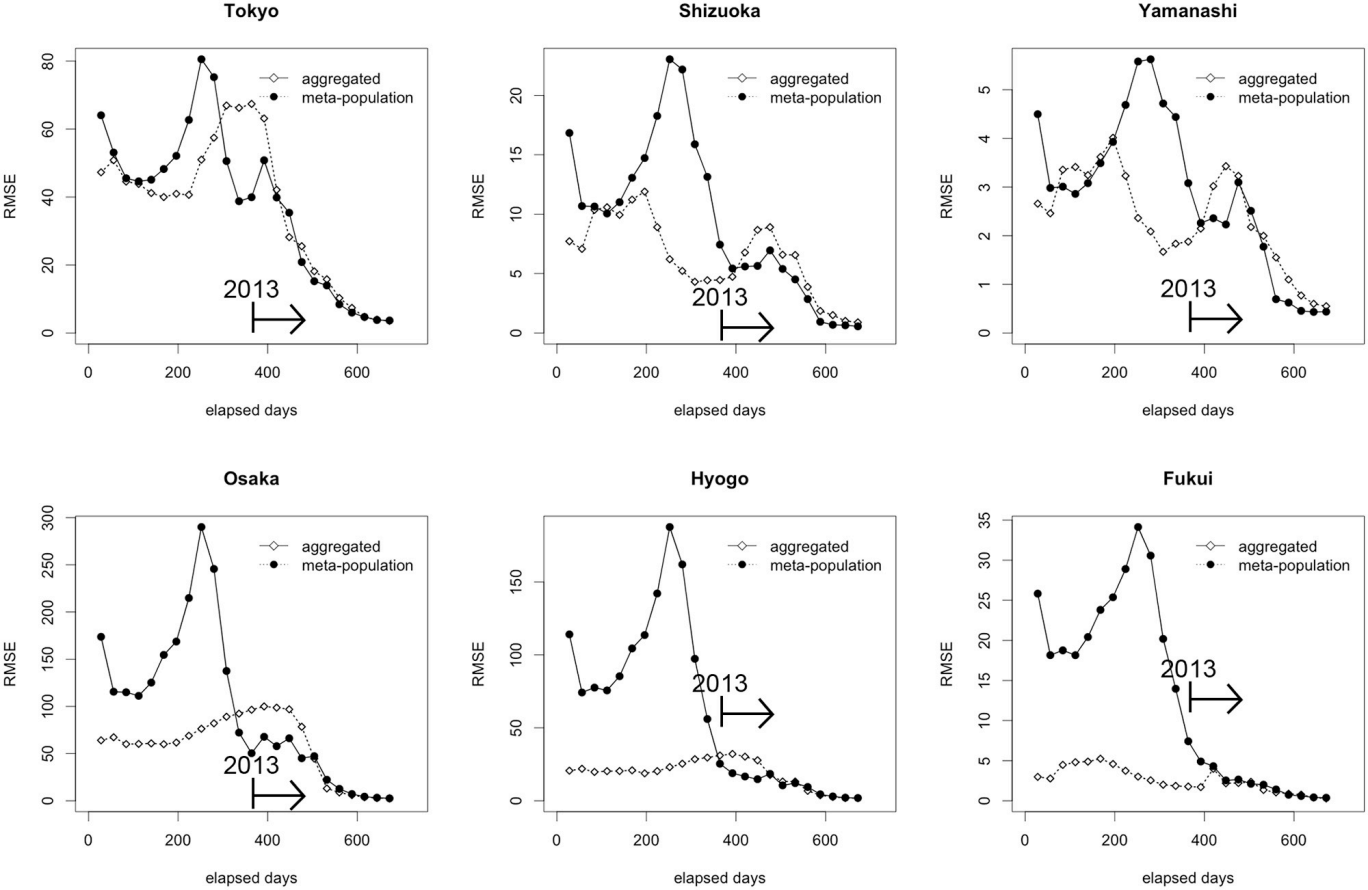
Comparison of the forecast error between metapopulation and homogeneous models. The horizontal axis measures the date on which forecasting was made. The vertical axis measures the root mean square error (RMSE), representing error value. For the homogeneous model, the SIR model without spatial structure was fitted to the dataset for all of Japan. Subsequently, infected individuals were proportionately allocated to each prefecture by population size, so that the prefecture-specific forecast can be compared. Metapopulation was labeled as “meta-population,” while the stochastic SIR model was labeled as “aggregated.” Day 0 is 2 January 2012. Days are counted onward; thus, year 2013 starts on Day 365.

Using Eq (8), Fig 5 compares the abovementioned RMSE_i_ between our metapopulation model and the SIR model without spatial structure. In the latter model, case counts were allocated by the relative population size to each prefecture. Due to stochastic spatial transmission dynamics, the metapopulation model reflects spiky curves in each prefecture and its RMSEs were greater than that of the SIR model without spatial components (see S1 Text and S4, S5 and S6 Figs that fully compared models with Poisson and binomially distributed likelihood functions). Moreover, estimates of the 2012 metapopulation epidemic were not accompanied by substantial reliability. The metapopulation model appeared to be superior to the homogeneous model from the midst of the 2013 epidemic onward.

### Spatial vaccination

**Fig 6:**
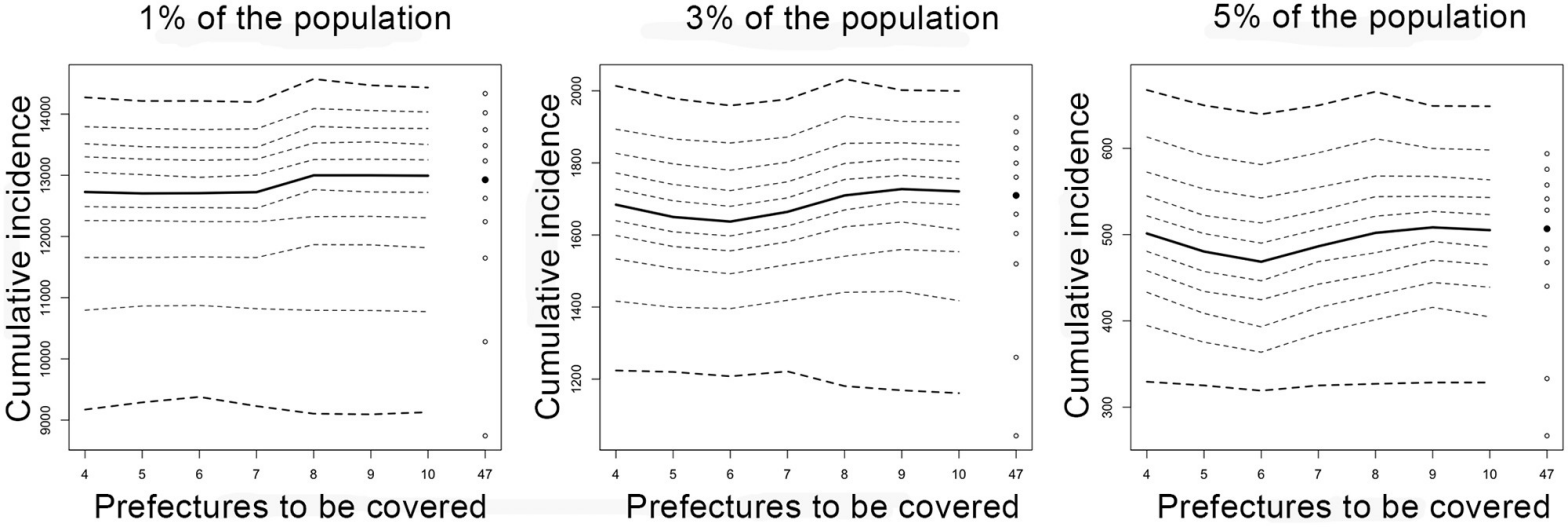
Predicted cumulative incidence by different spatial vaccination scenarios. Cumulative incidence is measured on the vertical axis. The spatial vaccination scenario assumes that we have enough vaccines to increases the immune fraction of the entire Japan by 1% (left), 3% (middle) and 5% (right), respectively, following each policy. Vaccination was assumed to take place in advance of the 2012 epidemic. The horizontal axis represents the number of prefectures to be covered by vaccination. For instance, the value of 4 indicates that vaccination is prioritized only for Tokyo, Kanagawa, Osaka and Aichi prefectures, i.e., the top four populous prefectures. The value of 47 indicates that vaccines are equally distributed to all prefectures. Median trajectory is represented by a continuous curve. Outer most curves are the 95% credible intervals. Other points are drawn using dotted lines for every 10th percentile point (i.e., 10th, 20th,…, 90th).

Fig 6 examines variations by different allocations of vaccination. The cumulative number of infected individuals slightly varied with the spatial extent of vaccination. When the vaccination coverage was 3% or 5%, the minimum cumulative count was achieved by allocating vaccines to the top six populous prefectures. While such minimum is identified, dashed lines represent uncertainties surrounding initial condition (of infectious individuals and susceptible individuals) and parameter estimates, which are greater than variations by the choice of prioritizing prefectures to receive vaccination. Interestingly, equal distribution of vaccination yielded a skewed distribution of the cumulative incidence.

## Discussion

The present study employed a metapopulation epidemic model to evaluate the spatiotemporal dynamics of rubella from 2012 to 2013 in Japan. Using a piecewise constant model for transmissibility, the epidemic curve was approximated by multiple exponential growth curves. We devised a particle MCMC method for inference, and the estimation of parameters during 2013 was successful, while the reliability of parameter estimates in 2012 appeared to be limited. Reflecting these findings, the forecast in 2012 was accompanied by broad uncertainty and large RMSE values, while that in 2013 nicely contained observed future data within the 95% CrI prediction intervals. Assuming that a very small amount of vaccines were distributed in advance of the epidemic, the spatial vaccination scenario appeared to be potentially valuable for selectively vaccinating specific prefectures, however, greater uncertainty was introduced by initial conditions and stochasticity.

To our knowledge, the present study is the first to implement spatiotemporal modeling of the recent rubella epidemic in Japan. The epidemic motivated us to employ a metapopulation model because the epidemic was largely observed in urban cities, including Tokyo and Osaka. Thus, we devised a model to capture that type of prefectural pattern, which indeed enabled us to yield the spatiotemporal forecast and examine potential usefulness of spatial vaccination. While the predictive performance was not necessarily substantial due to piecewise constant modeling with crude intervals as applied to the multimodal epidemic curve, especially in 2012, the forecast with narrower uncertainty bounds was obtained for the greater epidemic in 2013. Indeed, the error bound as measured by RMSE for the metapopulation model yielded better values than the homogeneous model in 2013.

Moreover, while it was accompanied by broad uncertainty, spatial vaccination scenarios indicated that vaccinating the top six populous prefectures (i.e., Tokyo, Kanagawa, Osaka, Aichi, Saitama and Chiba) could potentially be more effective than random allocation, motivating additional studies on this subject. Mechanistically, the model was accompanied by two technical issues that we aim to explicitly address in future study. First, the choice of initially infectious individuals was made by randomly generating initially infectious individuals in a specific prefecture(s) according to the size of susceptible individuals across prefectures. This simulation setting mirrors our assumption that the risk of importation in each prefecture is determined by the size of susceptible individuals. Nevertheless, to adhere to reality, imported cases would more frequently arise in urban prefectures than rural prefectures; thus, our recommendation to vaccinate urban locations might have been more conservative than the actual dynamics. Second, we assumed that the fraction susceptible was uniformly distributed across prefectures due to data limitations. Under this assumption, vaccinating urban prefectures due to selective vaccination could generate a susceptible pocket in rural prefectures, which could lead to an outbreak. Spatial heterogeneity in susceptibility could act as the key to discriminating the metapopulation model from homogeneous models, and our approach was unable to capture that aspect. It should be remembered that the susceptible pocket was observed mainly among males aged 35–49 years in Japan [19,20], which we intend to address in the forthcoming study with age structured model. The fraction of this “high risk” subpopulation may vary by prefecture and heterogeneous contact patterns would also differ. Again, urban prefectures, in reality, may be at greater risk than our simulations.

Other technical limitations that we cannot address in the present study must be emphasized. First, estimated transmissibility indicated the presence of seasonality, however, increasing and decreasing elements of the epidemic were not accompanied by explicit underlying causal mechanisms (i.e., why it increased and decreased). The eventual end of the 2013 epidemic, while leaving a substantial number of susceptible individuals [19], indicates the presence of seasonality. Second, chronological age structure was ignored for simplicity, however, the epidemic was actually aggregated among adult cases and may have been highly dependent on age-specific susceptibility and contact. A fully structured model with space and age is part of our ongoing future work. Third, while capturing spatial transmission, the spatial unit rested on a total of 47 prefectures in Japan and a micro-geographic scale to generate clusters was discarded. Fourth, due to data limitations, intra-annual variations in human movement, demography and other factors were not taken into account. Lastly, while our application was motivated by the 2012–2013 rubella epidemic in Japan, we did not examine CRS in relation to vaccination practice [3–8,21–26].

Despite numerous tasks for further improvements as described above, we believe that our exercise has successfully corresponded to our motivation in the first instance to capture spatiotemporal dynamics of rubella from 2012 to 2013 in Japan, which tended to be concentrated in urban prefectures. Additionally, by collecting more precise datasets including age dependency and susceptibility, we aim to devise a further improved model.

## Supporting information

S1 Text Supplementary text. Parameter estimation using parallel tempering and choice of observation model.
(DOCX)

S1 Fig Trajectory of MCMC chains with annealing temperature of 32. The figure shows how MCMC chains evolved for each parameter *R*_*g*,*s*_ (*g* = 1 or 2, *s* = 1,..5 or 6). Visual inspection indicated that only parameters related to 2013 (i.e., *R*_1,4_, *R*_1,5_, *R*_1,6_, *R*_2,4_ and *R*_1,5_) reach the state of convergence. The judgment was consistent with GRC-based checking if a looser condition (GRC < 2) than a conventional one (GRC < 1.1) is employed.
(PNG)

S2 Fig Forecasting rubella epidemic in 2012 and 2013, Japan. The antilogarithmic scaling of vertical axis of Fig 4 in the main text. Forecasts with 95% prediction intervals for six prefectures are shown. Six prefectures with different population sizes were selected. Bars measure the weekly number of reported cases. For the 2012 forecasting, we repeated the filtering process up to 112th day (when an exponential growth in the first year begins) and repeated the prediction process up to the end of the epidemic. Mean and 95% credible intervals (CrIs) in filtering process are displayed by solid curves and gray shaded areas, respectively. For the 2013 forecasting, the filtering process was continued up to 308th day on which an exponential growth in the second year began.
(TIF)

S3 Fig Procedure for estimation of transmissibility coefficients using parallel tempering. (TIF)

S4 Fig Comparison of variances between binomial and poisson distributions. Each panel shows a posterior distribution of Δ(*S*_*t*_ → *I*_*t*+1_) given *J*_obs_ (*J*_obs_ = 1, 10 or 50), assuming an uninformative flat prior and a likelihood function that follows either binomial (dashed line) or Poisson (filled line) distribution functions.
(TIF)

S5 Fig Forecasting rubella epidemic in 2012 and 2013, Japan, employing a binomial-type observation model. Similar plots to Fig 4 in the main text except that the observation model is replaced with a binomial distribution. Antilogarithmic scale is used to show the number of cases on vertical axis.
(TIF)

S6 Fig Comparison of the forecast error between metapopulation and homogeneous models with binomial-type observation model. Similar plots to Fig 5 in the main text except that the observation model is replaced with a binomial distribution.
(TIF)

S1 Table Epidemic data of rubella from 2012–13, Japan. (XLSX)
