# Supplementary material for: Reconstructing the transmission dynamics of rubella in Japan, 2012-2013
Source: PLoS One. 2018 Oct 17;13(10):e0205889. doi: 10.1371/journal.pone.0205889 (PMC6192647; doi:10.1371/journal.pone.0205889)
Supplement: S1 Text — Parameter estimation using parallel tempering and choice of observation model. (DOCX) [file pone.0205889.s001.docx]

**S1 Text. Supplementary text**

**Parameter estimation using parallel tempering and choice of observation model**

**Parameter estimation using parallel tempering**

This section is devoted to describe why we have employed the parallel tempering technique for the estimation of parameters (*β*)*_g,s_* that governed the piecewise constant model for transmissibility coefficients. As for general background knowledge on MCMC techniques, we refer, for example, [S1] at the bottom of this text.

The key mechanism that a Metropolis-type algorithm gives Monte Carlo estimation of a likelihood is to sometimes accept a parameter setting which is worse than what we have before iterative estimation. More explicitly, a transition from *θ* (better) to *θ′* (worse) is allowed with a probability *L*(*θ′*)/*L*(*θ*). This simple mechanism may not work when the magnitude of *L* is very large due the employed model that involves many latent variables. In the case of our model, the typical value of log *L* was 10,000 and the change of log *L* was around 1,000, then the acceptance probability of a proposed setting was exp(−1000) ≈ 5 × 10^−435^. Virtually all proposals toward “worse” setting is rejected: A Metropolis algorithm works just as a hill climbing, which does not work unless we have a very good initial guess. In addition, a MCMC trajectory frequently dives into a local maximum due to a similar mechanism.

The so-called simulated annealing technique mitigates this type of problem. Let us consider a function *L*(*θ*)^1^*^/T^* for *T* > 1 as a likelihood function instead of the original one and apply a Metropolis algorithm. Because *L*(*θ*)^1^*^/T^* has a wider tail than the original (note that *L*(*θ*)^1^*^/T^* tends to a uniform, as *T* → ∞), a Metropolis algorithm accepts many “worse” parameter settings which would be rejected under *L*(*θ*). The parameter *T* that controls how large the tail is widened is called (virtual) temperature. After the MCMC trajectory has reached around the “mountain” including the optimal point, by gradually reducing *T* toward unity, we may reach the optimal point.

A parallel tempering (or replica exchange) method enables us to evaluate the likelihood function under the assistance of the aforementioned annealing mechanism. The parallel tempering that we have employed here is formally viewed as a Metropolis-type algorithm where the original likelihood is to replace with a product of likelihoods that is widened with *n* different temperatures, i.e.,

$$L(\Theta)=L(\theta_{1})^{1/T_{1}}\times\cdots\times L(\theta_{n})^{1/T_{n}}.$$

We easily see that the original likelihood function is obtained as a marginal of this augmented likelihood. Therefore, if we have samples$(\Theta^{(i)})_{1\leq i\leq N}=(\theta_{1}^{(i)},\cdots,\theta_{n}^{(i)})_{1\leq i\leq N}$, which follows *L*(Θ), then we obtain the samples following to *L*(*θ*) by just keeping those corresponding to *T* = 1, $(\theta_{1}^{(i)})_{1\leq i\leq N}$, and ignoring the rest. In algorithmic level, Metropolis steps for **** (Θ) consists of two operations which is alternately carried out: usual Metropolis steps for each temperature and stochastic swapping of samples between two different temperatures. In fact, these two operations construct Metropolis steps for **** (Θ). The first operation is from, where Θ [*θi* ← *θi*′ ] denotes substitution *θi* in Θ with *θi*′. The second operation is explicitly defined as accepting the new state Θ [*θ_i_* ↔ *θ_j_* ], given by swapping between the present state and randomly chosen state with the probability

The entire procedure in our model is summarized in S3 Fig.

# **Choice of observation model**

In our study, the observation noise—discrepancy between the real data and model—are derived from the fact that not all patients are reported. For simplicity, if we accept an assumption that unreported cases randomly appear with an equal probability (say *p* here), we should employ a binomial distribution as that the distribution to which the observation noises follow. However, it is frequently the case that additional datasets (e.g. temporal change of seroprevalence) is necessary to explicitly estimate the probability *p* along with other parameters. Rather than doing so, we instead employed a Poisson distribution as the observation model in the main text, based on the approximation of Binom (*n*, *p*) ≈ Pois (*λ*) with *λ* = *np*. This approximation has not been sufficiently justified, because, though concrete value of *p* is unknown, it seems to be rather high in the current public health situation in Japan.

We then examine to what extent the choice of observation model (i.e. binomial or Poisson) is influential on our forecasting results by re-doing prediction experiments, replacing Poisson by binomially distributed observation model. Concretely speaking, we repeat experiment that was performed to draw Figs. 4 and 5 in the main text, using the following alternative likelihood

. (S1)

Division by *p*_0_ in the number of trial is for keeping the mean equal to the Poisson-distributed model. Discrepancy between binomial and Poisson, namely overestimation of the variance due to Poisson approximation, may become remarkable as *p* approaches to unity.

S4 Fig. shows a comparison of variances of posterior distributions between binomial and Poisson in typical scales of counts (*J* = 1, 10, and 50) that correspond to our exercise with *p* = 0.1, 0.5 and 0.9. According to visual inspection, the variance is not overestimated by Poisson compared with binomial for *p* < 0.5, while doubled or further increased if *p* > 0.9. We carry out experiments below, setting *p* = 0.9 as a typical situation where the discrepancy between Poisson and binomial distributions is likely seen.

S5 Fig. compares the observed data and predictions since 16th week (top panel) and 44th week (bottom panel), in a similar fashion to Fig. S2 (antilogarithmic version of Fig. 2) in the main text. The prediction since 16th week shows that mean predicted trajectory was similar to that of the Poisson model, but the variance was rather small and the confidence intervals in 2013 did not contain the empirical data, as expected from a simple comparison done in S4 Fig. Interestingly, the binomial distribution seems to be reluctant to catch up the data. Given empirical data up to 44th week, the Poisson-type model adjusts the prediction downward so as to match the peak of 2013 in scale (still with some overestimation of uncertainty), while the binomial likelihood failed to adjust even in scale (3-5 times larger than data around the peak). This result is somewhat paradoxical: the more a model tries to appropriately capture the data generating process, the poorer the performance of prediction would be. In addition to this particular finding, without Poisson(n+1) assumption, our implementation was not always led to successful convergence even after huge computational demand. Given these results, we decided to employ the Poisson distribution as the likelihood function in the main text.

As an empirical illustration, S6 Fig. compares RMSE*_i_*_,*|_*_τ_* as a function of τ (to which time point empirical data are available) between the metapopulation model and the SIR model without spatial structure. General temporal variation is similar to the Poisson version: the metapopulation model yielded much greater error in 2012 and comparable one in 2013. The binomial-type observation model generally yielded larger RMSE values than the Poisson-type.

In summary, choice of observation model, binomial or Poisson, did not have a profound impact on the time-dependent RMSE. Overestimation of the variance of posterior distributions may have been induced by Poisson observational model, and strictly speaking, analytically more appropriate approach might have been to use binomial distribution. However, the simple Poisson model captured the overall temporal dynamics of rubella from 2012-13.

**Supplementary references**

S1. Gamerman, D. (1997) Markov Chain Monte Carlo, Chapman & Hall/CRC.
