# Supplementary figures and images for: Reconstructing the transmission dynamics of rubella in Japan, 2012-2013
Source: PLoS One. 2018 Oct 17;13(10):e0205889. doi: 10.1371/journal.pone.0205889 (PMC6192647; doi:10.1371/journal.pone.0205889)

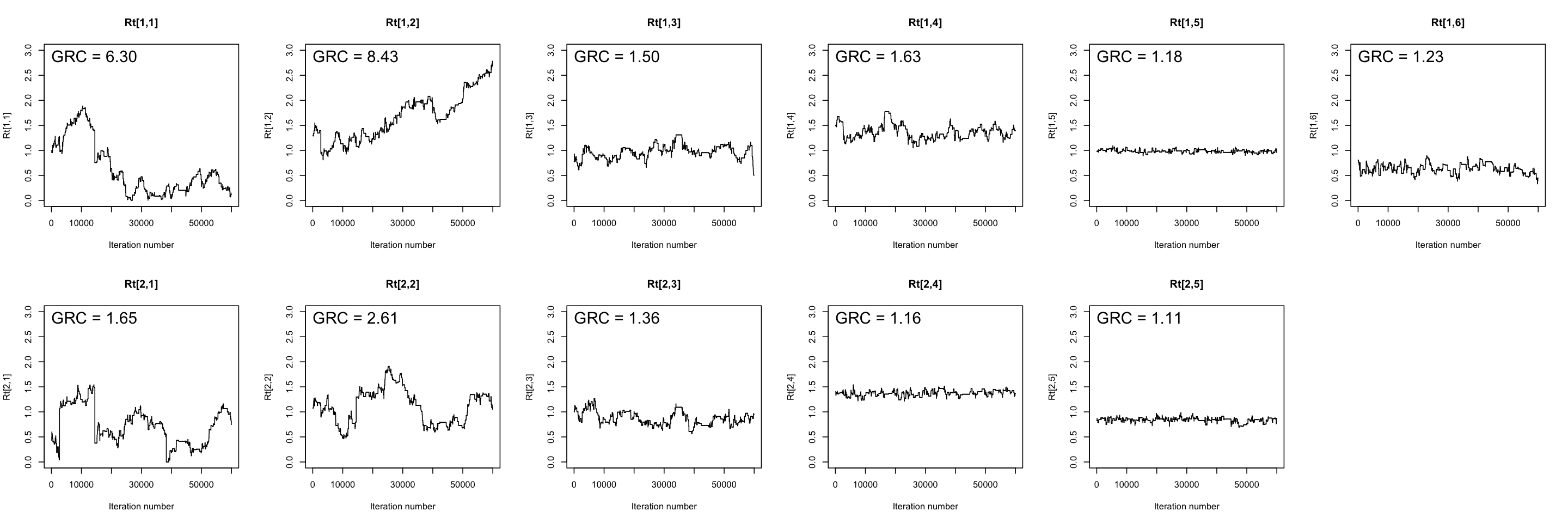

Supplement: S1 Fig — The figure shows how MCMC chains evolved for each parameter Rg,s (g = 1 or 2, s = 1,..5 or 6). Visual inspection indicated that only parameters related to 2013 (i.e., R1,4, R1,5, R1,6, R2,4 and R1,5) reach the state of convergence. The judgment was consistent with GRC-based checking if a looser condition (GRC < 2) than a conventional one (GRC < 1.1) is employed. (PNG) [file pone.0205889.s002.png]

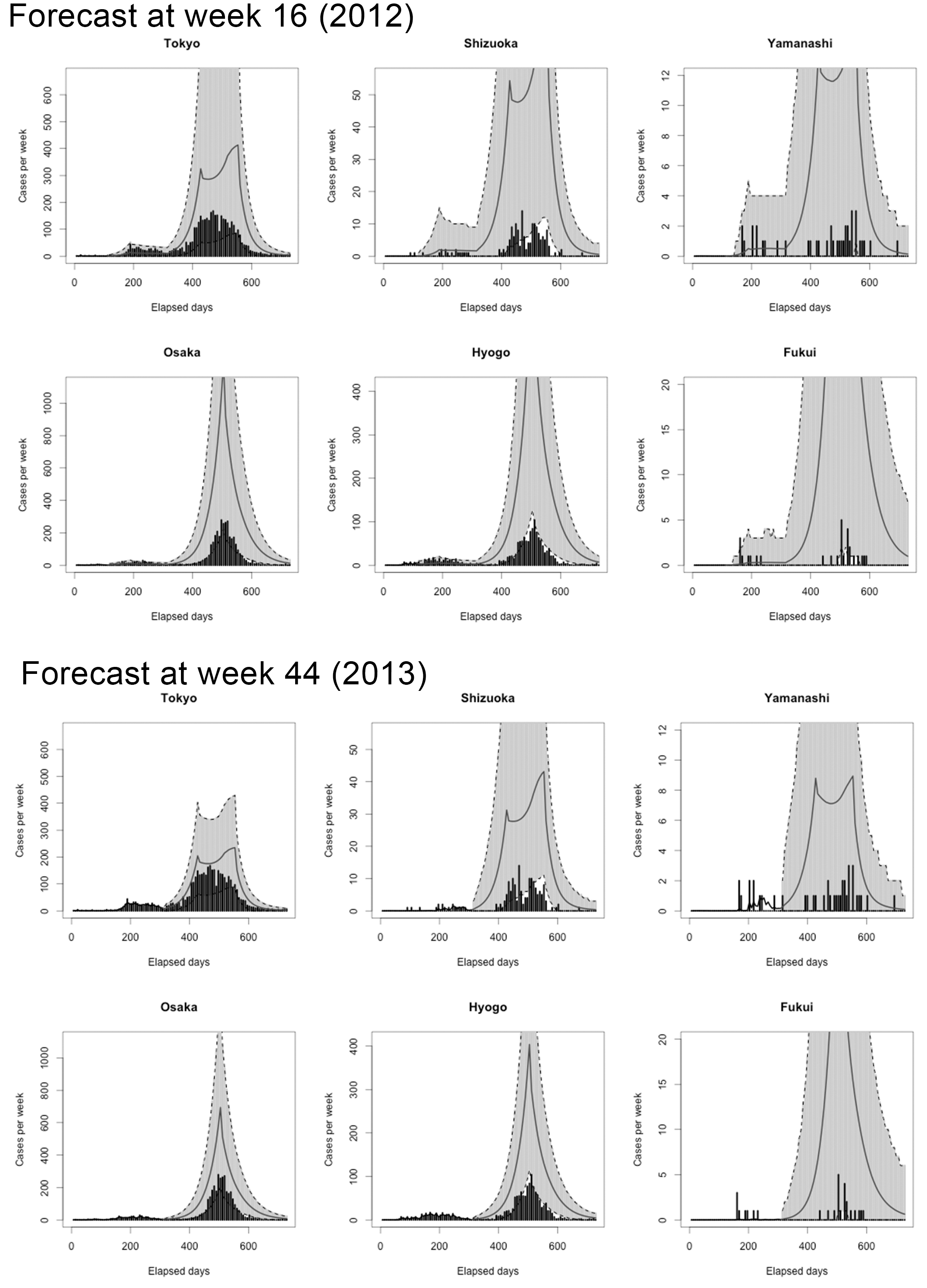

Supplement: S2 Fig — The antilogarithmic scaling of vertical axis of Fig 4 in the main text. Forecasts with 95% prediction intervals for six prefectures are shown. Six prefectures with different population sizes were selected. Bars measure the weekly number of reported cases. For the 2012 forecasting, we repeated the filtering process up to 112th day (when an exponential growth in the first year begins) and repeated the prediction process up to the end of the epidemic. Mean and 95% credible intervals (CrIs) in filtering process are displayed by solid curves and gray shaded areas, respectively. For the 2013 forecasting, the filtering process was continued up to 308th day on which an exponential growth in the second year began. (TIF) [file pone.0205889.s003.tif]

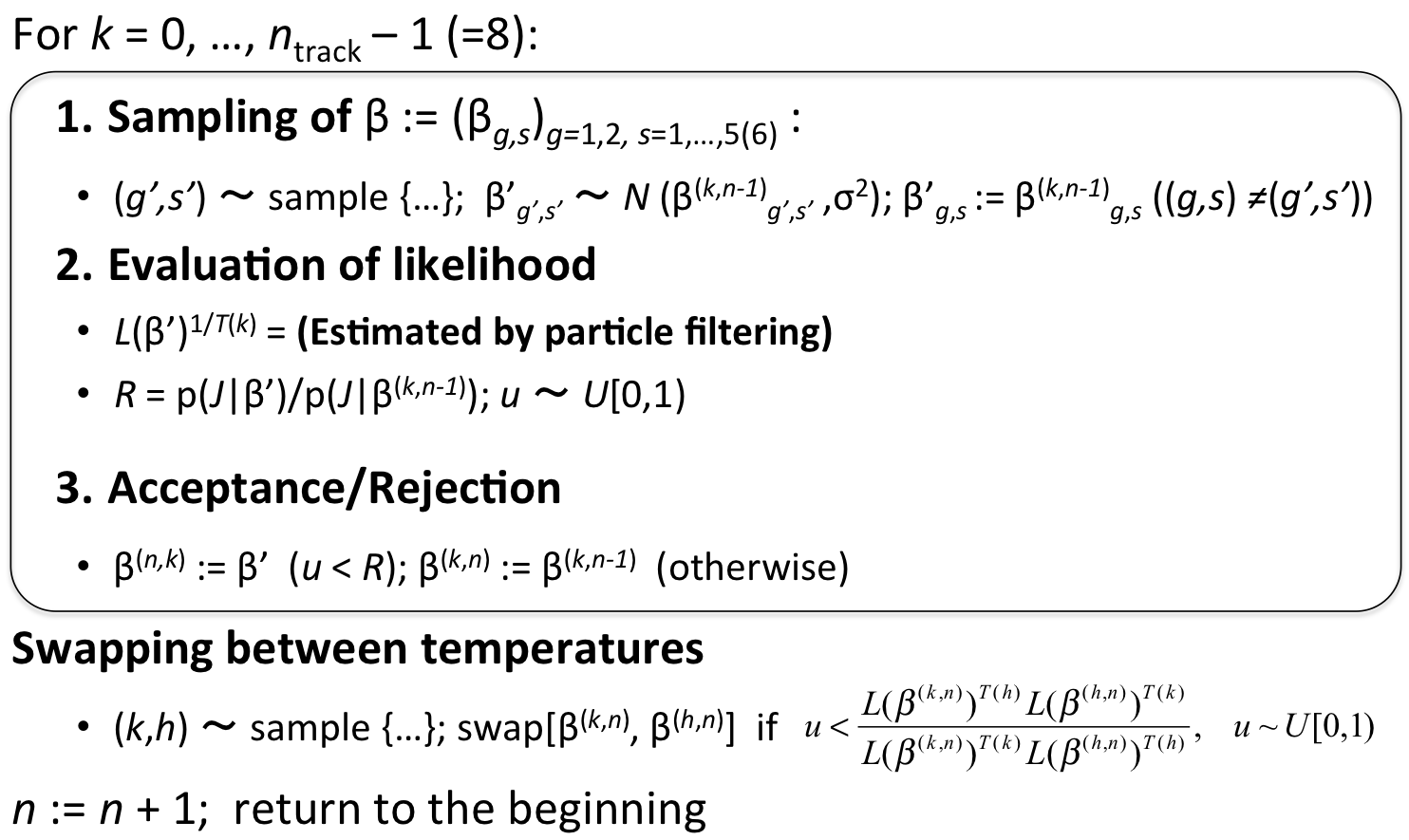

Supplement: S3 Fig — (TIF) [file pone.0205889.s004.tif]

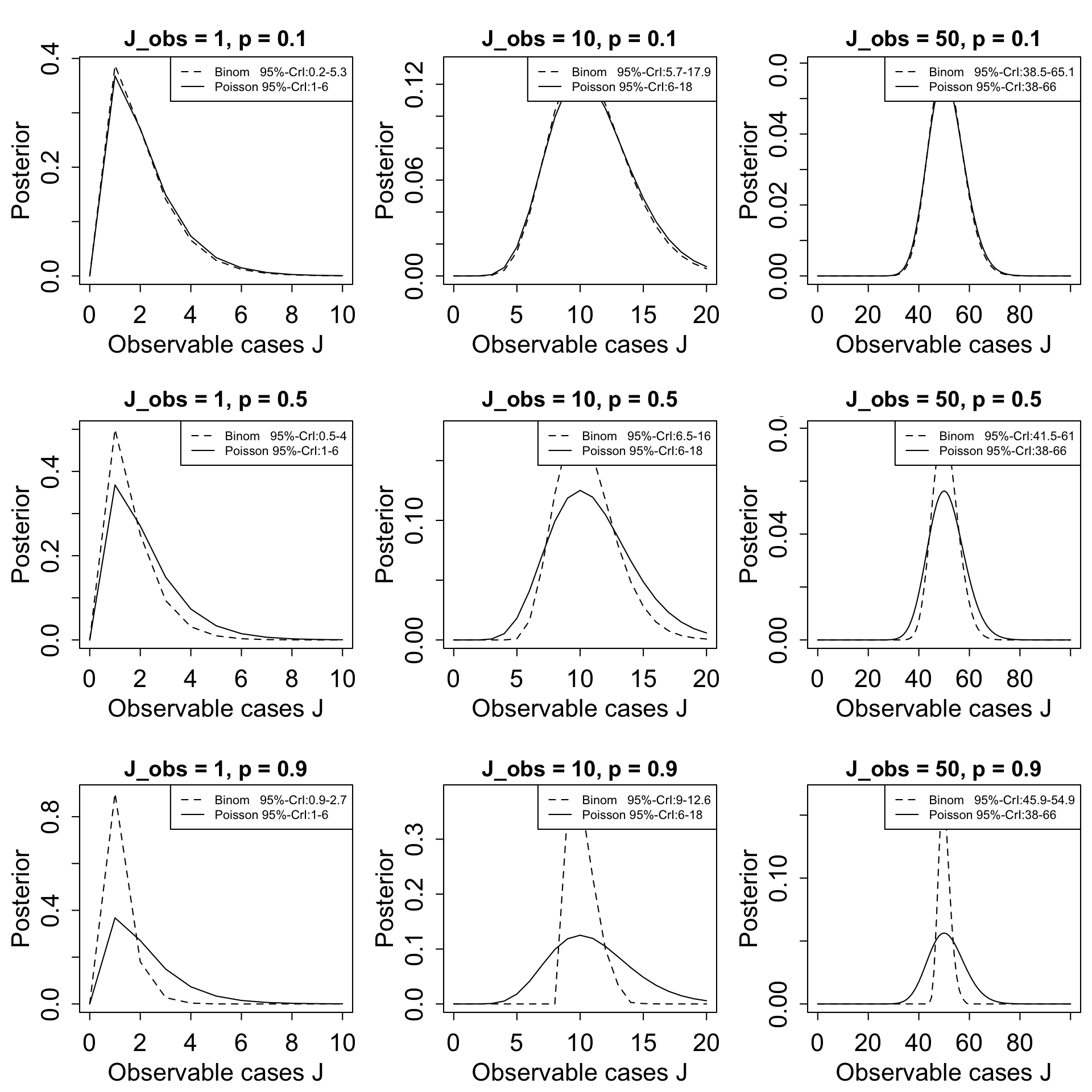

Supplement: S4 Fig — Each panel shows a posterior distribution of Δ(St → It+1) given Jobs (Jobs = 1, 10 or 50), assuming an uninformative flat prior and a likelihood function that follows either binomial (dashed line) or Poisson (filled line) distribution functions. (TIF) [file pone.0205889.s005.tif]

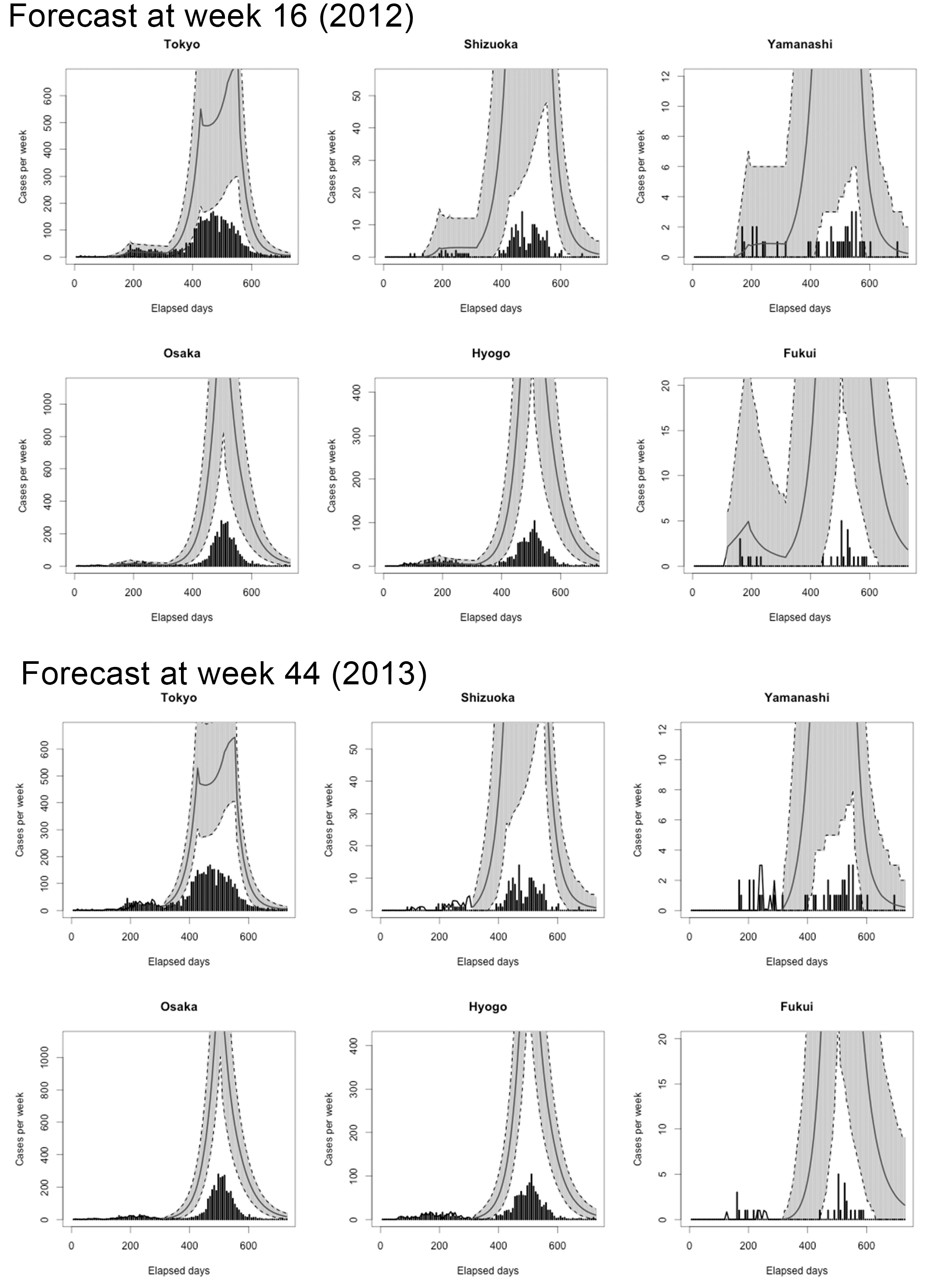

Supplement: S5 Fig — Similar plots to Fig 4 in the main text except that the observation model is replaced with a binomial distribution. Antilogarithmic scale is used to show the number of cases on vertical axis. (TIF) [file pone.0205889.s006.tif]

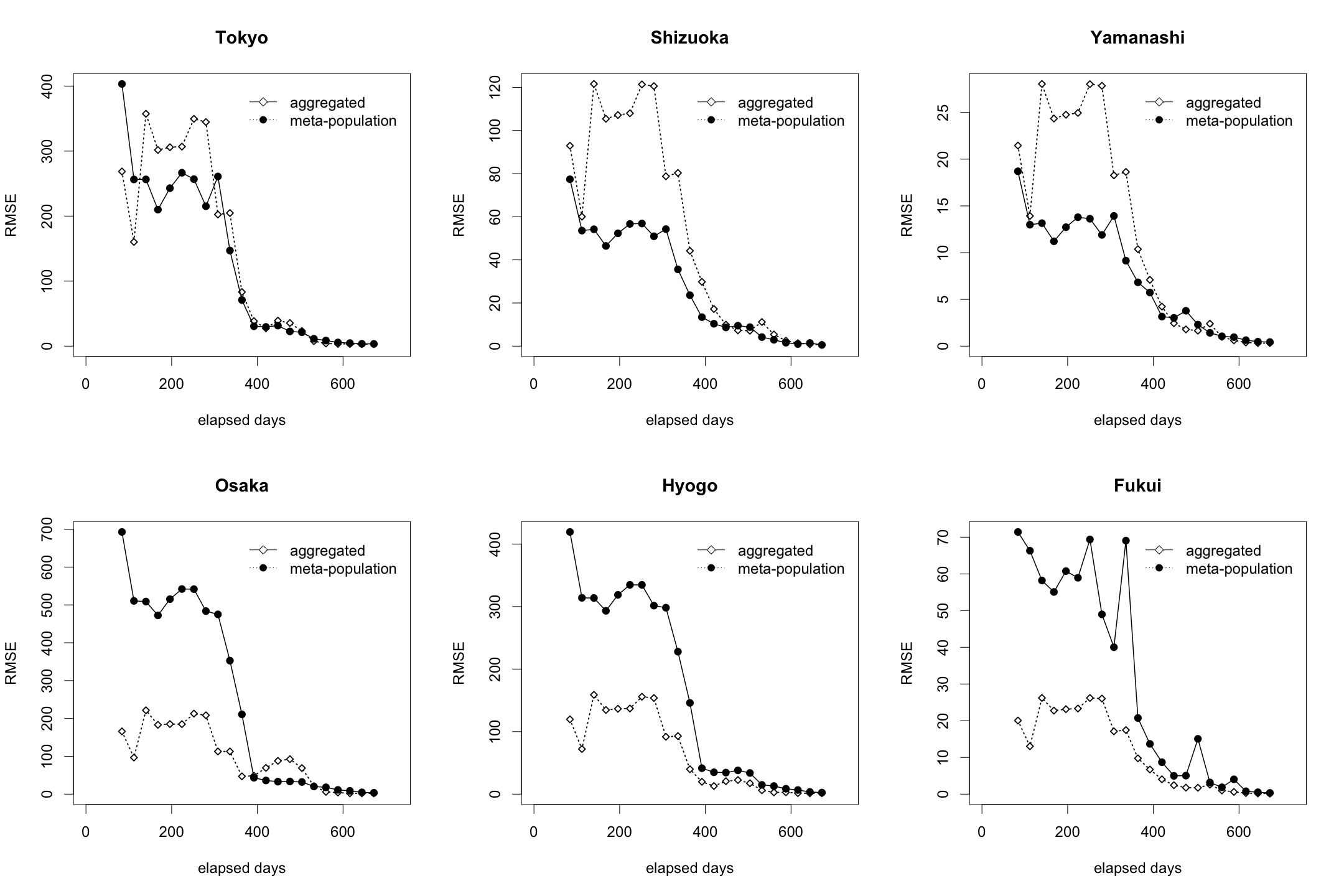

Supplement: S6 Fig — Similar plots to Fig 5 in the main text except that the observation model is replaced with a binomial distribution. (TIF) [file pone.0205889.s007.tif]
